# Using the Photoluminescence Color Change in Cesium Lead Iodide Nanoparticles to Monitor the Kinetics of an External Organohalide Chemical Reaction by Halide Exchange

**DOI:** 10.1021/acsnanoscienceau.3c00026

**Published:** 2023-09-07

**Authors:** Tennyson L. Doane, Kevin J. Cruz, Tsung-Hsing Chiang, Mathew M. Maye

**Affiliations:** Department of Chemistry, Syracuse University, Syracuse, New York 13244, United States

**Keywords:** cesium lead iodide, colorimetric, perovskite, assay, sensing, organohalide

## Abstract

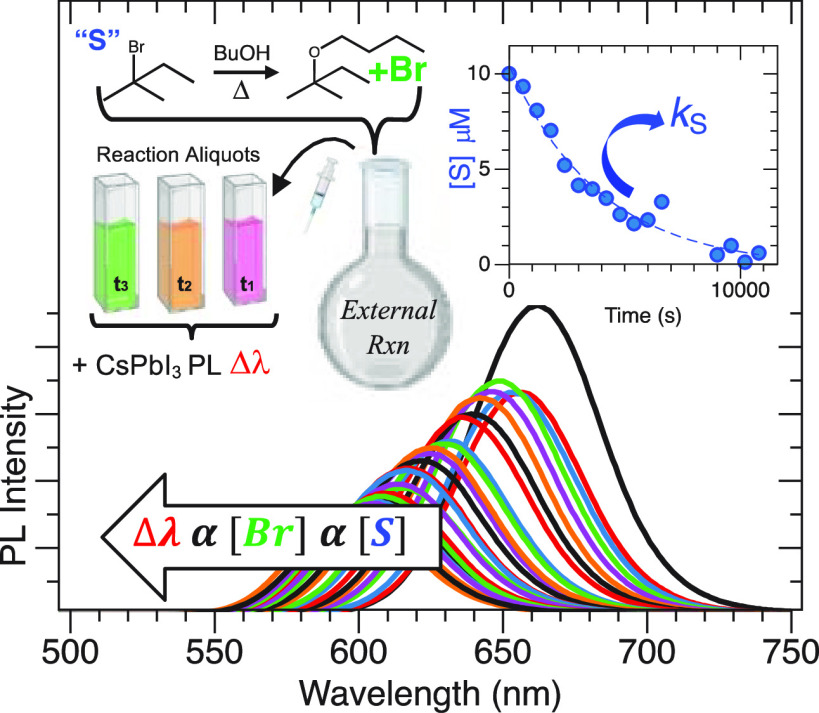

In this work, we demonstrate a photoluminescence-based method to monitor the kinetics of an organohalide reaction by way of detecting released bromide ions at cesium lead halide nanoparticles. Small aliquots of the reaction are added to an assay with known concentrations of CsPbI_3_, and the resulting Br-to-I halide exchange (HE) results in rapid and sensitive wavelength blueshifts (Δλ) due to CsPbBr_*x*_I_3*–x*_ intermediate concentrations, the wavelengths of which are proportional to concentrations. An assay response factor, *C*, relates Δλ to Br^–^ concentration as a function of CsPbI_3_ concentration. The observed kinetics, as well as calculated rate constants, equilibrium, and activation energy of the solvolysis reaction tested correspond closely to synthetic literature values, validating the assay. Factors that influence the sensitivity and performance of the assay, such as CsPbI_3_ size, morphology, and concentration, are discussed.

## Introduction

All inorganic cesium lead halides (CsPbX_3_, X = Cl, Br, and I) and hybrid methylammonium lead halides (MAPbX_3_) are important functional materials^1−6^ that can be synthesized as nanoparticles with a variety of compositions, crystal structures, and morphologies.^7−18^ These materials have broad absorption energies and narrow photoluminescence (PL), the wavelength of which is sensitive to halide and mixed halide stoichiometry. As ionization energies decrease from F^–^ > Cl^–^ > Br^–^ > I^–^, the resulting highest occupied molecular orbital (HOMO) contribution to the valence band of CsPbX_3_ results in band gap energy (*E*_g_) decreases, with, for example, a CsPbBr_3_ nanoparticle emitting at higher energies than CsPbI_3_.^19^ The precise emission wavelength is further a function of quantum confinement (*e.g.*, size and morphology). A novel property of these materials is the rapid ion exchange (IE) that can occur without altering the morphology or size, with halide exchange (HE) in particular serving as a powerful way to fine-tune PL emission and absorption postsynthesis.^20−25^ While IE is also observed in chalcogen quantum rods^26^ and quantum dots,^27^ the HE in CsPbX_3_ is more sensitive to the thermodynamics of the ternary ionic lattices,^7,28,29^ the kinetics of nonequilibrium conditions,^30^ more labile ligand capping,^31^ and especially high ion vacancy concentrations.^32−34^ The use of HE in PL-based sensing has been explored for the detection of halomethanes,^35^ as well as detection of halides in oils^36^ and sweat.^37^ In addition, organohalides have served as halide precursors in CsPbX_3_ syntheses, including bromobenzene^38^ and benzoyl halides.^39^

In this study, we explore the potential of using HE between I^–^ within CsPbI_3_ and Br^–^ ions released as a product of an external organohalide reaction. CsPbI_3_ has been synthesized in a number of ways,^7,16,17^ and HE with Br^–^ ions has been shown previously by others using an array of Br^–^ sources, as well as by our lab when using TOABr.^30^ The result of this study is a colorimetric assay that is sensitive to target concentration not by PL intensity changes but instead by significant color changes in the form of wavelength blueshifts up to ∼120 nm. The kinetics, equilibrium, and activation energies of the organic reaction were extracted from the color changes and match values measured using conventional approaches, indicating the effectiveness and sensitivity of the assay.

## Experimental

### Chemicals and Reagents

Lead(II) iodide (PbI_2_, 99%), cesium carbonate (Cs_2_CO_3_, 97%), 1-octadecene (ODE, 90%), oleic acid (OAc, 90%), oleylamine (OAm, 70%), methanol (MeOH, 99.8%), 1-butanol (BuOH, 99.8%), methyl acetate (MeAc, 99%), potassium bromide (KBr, 99%), hydrobromic acid (HBr, 48%), and 2-bromo-2-methylbutane (S, 99%) were purchased from Sigma Aldrich. Hexanes (Hx, 95.5%) and acetone (Ac, 99.5%) were manufactured by BDH. Dimethyl sulfoxide (DMSO, 99.9%) and potassium hydroxide (KOH, 88.1%) were purchased from Fisher Scientific, and ethanol (EtOH, 200 proof) was manufactured by Pharmco-AAPER. All chemicals were used without further purification.

### CsPbI_3_ Synthesis and Purification

The synthesis of the CsPbI_3_ nanoparticles followed a previously published protocol.^40^ Briefly, 84 mg of PbI_2_ was mixed with 5 mL of octadecene and was heated first under vacuum at 120 °C for 40 min and then placed under argon (Ar); then, 0.5 mL of both neat OAm and neat OAc was injected into the solution. The reaction was allowed to mix until PbI_2_ was dissolved, yielding a faint yellow solution. The temperature (*T*) was then raised to 140 °C and allowed to equilibrate. Next, a premade solution of 0.125 M cesium oleate (Cs_2_CO_3_ dissolved in OAc and degassed) was heated to ∼80 °C, and then, a 0.4 mL aliquot was rapidly injected into the PbI_2_ solution. Upon injection, the solution turned dark red, indicating the nucleation and growth of OAm- and OAc-capped CsPbI_3_, and the reaction was quenched by removing the vessel from the heating mantle and cooled carefully in a water bath. Upon cooling, multiple 1 mL aliquots were removed, stored under Ar, and refrigerated. Before use, these samples were first centrifuged at 5000 rpm and 1844*g* for 1 min to remove large aggregates. The Hx-rich supernatant was transferred and concentrated to ∼0.2 mL using either a rotavap or N_2_ stream before 1.5 mL of BuOH or MeAc^41^ was added in an Eppendorf tube. The tubes were then vortexed before being centrifuged at 10,000 rpm (7378*g*) to fine pellets, dried under Ar, and resuspended in 1.0 mL of Hx. The concentrations of the final stock solutions were determined spectroscopically using an estimated extinction coefficient (ε) of 6.4 × 10^6^ M^–1^ cm^–1^ at 425 nm.^20^

### Solvolysis Reaction and CsPbI_3_ Assay

In a typical experiment, a 25 mL four-neck round-bottom flask with a condenser was loaded with 10 mL of BuOH and heated to the target temperature. Next, 12.8 μL of 2-bromo-2-methylbutane (S) was added, yielding a 10 mM solution. During the reaction, a 100 μL aliquot was removed and immediately cooled. Next, 2.5 μL of this aliquot was injected into an Eppendorf tube or cuvette containing a 700 μL Hx solution of CsPbI_3_ and allowed to equilibrate for 3 min before PL measurements. Here, we note that the CsPbI_3_ concentration was independently determined spectroscopically before each aliquot was added to ensure consistency.

### Instrumentation

Optical characterization was performed on a Cary 50 Bio UV–vis spectrophotometer (Varian, Inc.), and photoluminescence spectroscopy was performed on a Cary Eclipse fluorescence spectrophotometer (Varian, Inc.). The excitation wavelength was 400 nm. The powder X-ray diffraction (XRD) measurements were performed using a D2 PHASER (Bruker, Inc.) with a Cu radiation source. Samples were prepared by drop-casting purified products on a zero-diffraction quartz holder or by addition of dried powders. Transmission electron microscopy (TEM) was performed on a JEM 1400 (JEOL, Inc.) operated at 120 kV using samples drop-cast onto carbon-coated Cu grids.

## Results and Discussion

**Figure 1:**
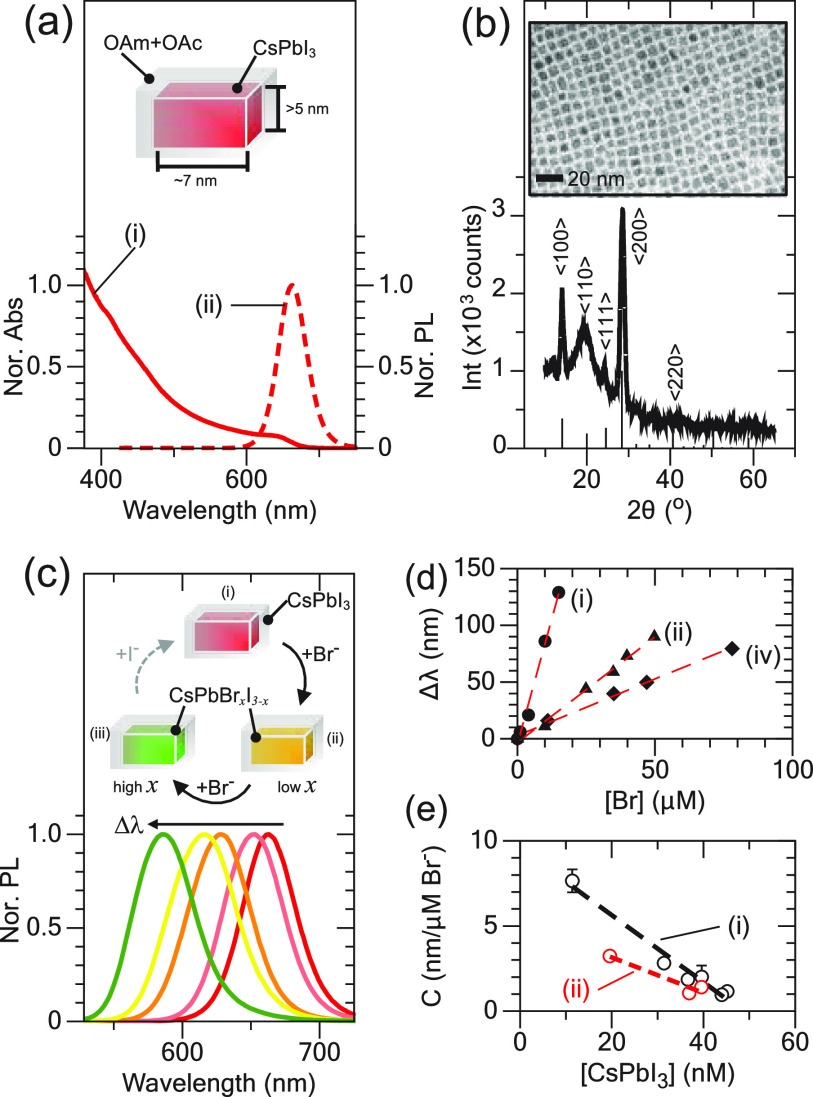
(a) Representative UV–vis (i) and PL (ii) of OAm- and OAc-capped CsPbI_3_ in hexane. (b) XRD of CsPbI_3_ with cubic reference patterns shown. Inset: TEM of CsPbI_3_. (c) HE-induced PL blueshift at increasing [HBr]. Inset: illustration of HE processes. (d) Calibration plot relating HE to Δλ at [CsPbI_3_] = 11 (i), 31 (ii), and 40 nM (iii). (e) Relationship between CsPbI_3_ with volumes of 650 (i) and 1100 nm^3^ (ii) and concentration with determined sensitivity parameter *C*.

Figure 1a shows a representative set of UV–visible absorbance (UV–vis, i) and photoluminescence emission (PL, ii) spectra for the all inorganic cesium lead iodide nanoparticles (CsPbI_3_) used in this study that were synthesized with oleylamine (OAm) and oleic acid (OAc) ligand capping and purified following reported methods.^33,40^ The first band edge absorption observed by UV–vis corresponds to the minimum edge length for CsPbI_3_ whose nanoform is typically platelets, which corresponds to a thickness of >5 nm for absorption at ∼640 nm. The corresponding PL emission wavelength was centered at 663 nm (λ_0_). Figure 1b shows the powder X-ray diffraction (XRD) and transmission electron microscopy (TEM) micrograph of CsPbI_3_, revealing a cubic crystal structure and square-plate-like morphology, with average edge lengths (*l*) of 8.5 ± 1.3 nm. Here, we note that CsPbI_3_ products purified with methyl acetate (MeAc) were more stable in both PL quantum yield (QY) as well as stoichiometry and size.^41^ Nonetheless, CsPbI_3_ was stored in dry solvents under nitrogen and used soon after synthesis, as older samples showed evidence of QY loss associated with CsPbI_3_ sintering and CsPb_2_I_5_ phases forming, as was revealed by TEM and shown in Figure S1. Such impurities are also indicated by the broad diffraction at ∼20°. The CsPbI_3_ concentrations in solution were estimated using UV–vis absorbance at 425 nm.^20,33^

When reacted with bromine ions (Br^–^), the CsPbI_3_ crystal structure undergoes halide exchange (HE) to form CsPbBr_*x*_I_3*–x*_ mixed halide crystals, which is driven in large part by high ion vacancies at the crystal surface after synthesis and CsPbBr_3_ having more favorable Goldschmidt parameters.^28,29,42^ Moreover, as *x* increases, so too does the material’s band gap (*E*_g_), and thus, a UV–vis and PL emission blueshift to a new wavelength (λ_*t*_) occurs. It is important to note that an opposite redshift occurs when CsPbBr_*x*_I_3*–x*_ is reacted with excess I^–^ sources, although that is not studied in this system.^31^ In this paper, we focus on monitoring the PL change, but it is important to note that similar results can be achieved using UV–vis wavelength changes.

Figure 1c illustrates the HE observation and shows calibration of the PL wavelength change, Δλ = λ_0_ – λ_*t*_, where λ_0_ is the initial PL and λ_*t*_ is the PL maximum at either a known Br^–^ concentration or aliquot sampling time. In this study, the CsPbI_3_ concentration was determined as described above, and the Br^–^ source for calibration was the known concentration of HBr dissolved in BuOH, although other Br^–^ sources can be used, such as tetraoctylammoniumbromide (TOABr).^30^ As shown in Figure 1c, increasing the Br^–^ concentration ([Br^–^]) causes a Δλ of ∼90 nm, and this response was found to be proportional to the total CsPbI_3_ concentration in solution ([CsPbI_3_]). Figure 1d shows the resulting calibration plot showing a linear correlation between Δλ and [Br^–^] for [CsPbI_3_] of ∼11 (i), 31 (ii), and 40 nM (iii).
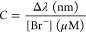
1

To better combine these three parameters, Figure 1e plots the so-called sensitivity factor (*C*) as a function of [CsPbI_3_], where *C* has units of nanometer shift per micromolar Br^–^ (Δλ (nm)/[Br^–^] μΜ), as shown in eq 1. For instance, a solution or assay using [CsPbI_3_] = 30 nM can expect a Δλ = 3.7 nm per μM of Br^–^, with a maximum Δλ of ∼120 nm (*i.e.*, complete exchange). The *C*-value also indicates that care must be made to add sufficient [CsPbI_3_] as to not rapidly saturate the ion exchange response (*i.e.,* low [CsPbI_3_] compared to high [Br^–^]). In the assay studies below, [CsPbI_3_] was kept between 20 and 40 nM, and the concentrations of the reactants in the reaction were adjusted accordingly. Further, we repeatedly found a linear Δλ trend up to what we estimated to be an *x* of ∼0.8 (*i.e.*, CsPbBr_0.8_I_0.2_, Δλ = 122 nm), at which point ion equilibrium between I^–^ and Br^–^ plays a more direct role. Finally, the HE was fast, and Figure S2 shows detailed PL spectra of the assay response over 200 s.

**Figure 2:**
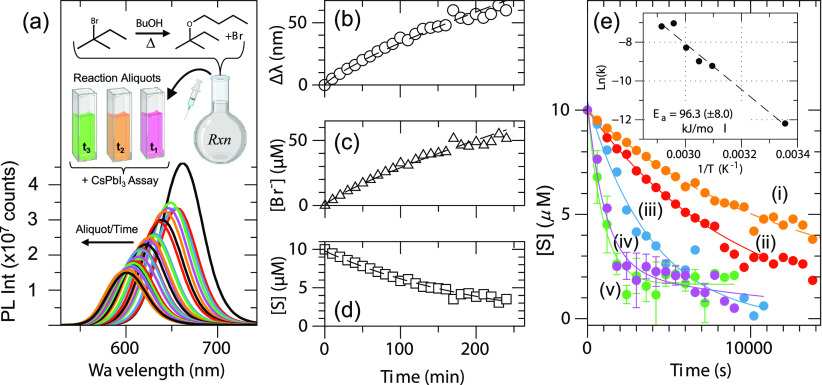
(a) PL monitoring of the CsPbI_3_ assay during the reaction. Inset: schematic illustration of the experiment. (b) Corresponding kinetic traces of Δλ. (c) Plot of the [Br^–^] increase over the reaction using *C* = 1.302 nm/μM. (d) Plot of [S] consumption over time using eq 2 and *D* = 126. (e) Kinetic traces of [S] *versus* time at *T* = 50 (i), 55 (ii), 60 (iii), 65 (iv), and 75 °C (v). Inset: corresponding Arrhenius plot. Fitting parameters and representative spectra are found in Tables S1 and S2 and Figure S4.

It was hypothesized that HE could be used as a sensitive colorimetric assay of Br^–^ in solution, particularly Br^–^ released during an organohalide reaction. Such an approach is illustrated in Figure 2a, where a solvolysis reaction between 2-bromo-2-methylbutane (S) and 1-butanol (BuOH) forms *tert*-amylbutylether (P), protons (H^+^), and Br^–^. This reaction is known to go to completion and have simple kinetics, making it a reasonable proof of concept. As the reaction occurs, 2.5 μL aliquots are sampled and then added to separate vials or cuvettes containing known [CsPbI_3_] and volume (700 μL), and the resulting Δλ is an indicator of S consumption, thus a proxy for kinetics.

Figure 2a shows the HE-based PL monitoring over the course of the solvolysis reaction, with reaction aliquots sampled every 10 min. The corresponding kinetic plot, Δλ plotted *versus* time, is shown in Figure 2b. Δλ can then be converted to [Br^–^] using *C* = 1.302 nm/μM Br^–^ at [CsPbI_3_] = 46 nM. From this, a kinetic trace is generated by conversion to [S] by:
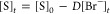
2where [Br^**–**^]_*t*_ is the assay-determined concentration and *D* is a dilution factor considering the aliquot and assay volumes. Figure 2d shows the reaction kinetic trace as a function of time. Control experiments showed negligible Δλ if S was added directly to a solution of CsPbI_3_, indicating that PL changes were the result of Br^**–**^ release during the organic reaction and not direct HE, as shown in Figure S3. We also note that the charge pair for Br^**–**^ is likely H^+^ from BuOH; however, it could also be a protonated oleylammonium (OAm^+^) ligand closely associated with the CsPbI_3_ organic capping by the time of HE.^31,33^

To show the utility of this assay, reactions at different temperatures were studied. Figure 2e shows the kinetic traces of aliquots sampled when the solvolysis reaction proceeded at *T* = 50 (i), 55 (ii), 60 (iii), 65 (iv), and 70 °C (v). Here, we note that the assay HE reaction occurred at room temperature, while aliquots were sampled from the hot reaction. The *T*-dependent kinetics as well as final concentrations or equilibrium can be observed. The equilibrium in particular shows a well-known phenomenon where the buildup of HBr promotes acid-catalyzed ether cleavage and regenerates S (reverse reaction), as has been reported for other tertiary ethers under anhydrous conditions.^43,44^ These results also act as reminders that only a small portion of the reaction medium is removed for each assay point. Aliquots from 65 (iv) and 75 °C (v) showed some variability in λ_*t*_, as shown in the error bars from repeating the assay three times. We attribute this variability, especially at early sampling times, to the higher [Br^–^] in those aliquots and likely nonuniform pipette-based mixing, resulting in varied CsPbBr_*x*_I_3*–x*_ compositions, leading to broad PL and sample-to-sample variability.

From these kinetic plots, the reaction rates were determined, which for an S_N_1 reaction like this solvolysis obeys a first-order rate law with respect to [S] by
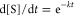
3where *k* is the apparent rate constant at *T*. The data in Figure 2e were fit to a single exponential decay (Table S1) from which *k* was calculated (Table S2). For reference, the predicted solvolysis rates based on literature values for activation energy (*E*_a_) of 103.3 kJ/mol and a pre-exponential factor (*A*) of 6.626 × 10^18^ are shown as dashed lines.^45^ At elevated *T*, the trends change due to acid-catalyzed tertiary ether cleavage, which is a bimolecular reaction with second-order kinetics and is governed by a slow R_3_C–OH^+^–R rearrangement.^46^ Assuming that the rearrangement is the slowest step, the kinetic rate relationship can be approximated in the steady-state condition as a pseudoreversible first-order reaction given as follows:
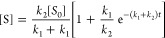
4where *k*_1_ is the rate for solvolysis of *S* to form P and *k*_2_ is the rate for HBr-mediated cleavage to regenerate [S]. Therefore, fitting the data in Figure 2e(i–iv) with a single exponential and rearranging terms, we find that *k*_1_ = 884.7 × 10^–6^ s and *k*_2_ = 174.6 × 10^–6^ s (see Tables S1 and S2). At 70 °C, Figure 2e(v), a biexponential rate function was the best fit, with a fast component of *k*_fast_ = 923.1 × 10^–6^ s and *k*_slow_ = 77.9 × 10^–6^ s, suggesting that the forward reaction becomes more competitive at 70 °C. Fitting the region between 0 and 3600 s with a single exponential and using eq 1, we find that *k*_1_ = 725.4 × 10^–6^ s and *k*_2_ = 162.22 × 10^–6^ s.^47,48^

Finally, these extracted temperature-dependent *k* values were used to calculate *E*_a_ for the solvolysis reaction, as shown in the inset of Figure 2e and fit using the Arrhenius relation in eq 5,
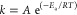
5which estimates *E*_a_ to be 96.3 ± 8.0 kJ/mol, a value that is close to the NMR-determined one of 103.3 kJ/mol,^45,49^ further validating that the PL response of CsPbI_3_ serves as an accurate measurement of the reaction progress. Here, we note that the lowest *T* point in the Arrhenius plot is taken from the literature.^45,49^ The discrepancy in values is likely the result of the variability of the assay at this early point and potentially from an imprecise ε used to estimate the CsPbI_3_ concentration.

Taken together, these results indicate that HE at CsPbI_3_ serves as a sensitive colorimetric way to detect Br^–^ ions released during a chemical reaction. While not the focus of this study, the sensitivity or detection limit of such an assay is expected to be low considering that it is PL-based and that CsPbI_3_ and other CsPbX_3_ (X = Cl, Br, and I) have high QY. Moreover, in this study, detection limits were not critical, as the chemical reaction occurred at high concentrations. However, precision and performance of the PL shift as well as peak broadening will be influenced by several morphological and stoichiometric factors. In the assay above, one size and morphology of CsPbI_3_ were used, but response will be related to these factors since they dictate the total amount of ions available for HE. In essence, larger sizes (*i.e.*, volumes) will require higher [Br^–^] to be exchanged with I^–^ to significantly alter the stoichiometry enough for a PL change. More studies are needed, but Figure 1e shows a measured *C* for CsPbI_3_ at two volumes, revealing a lower slope for larger sizes indicating less sensitivity, and Figure S5 shows a calculation of the relationship between the CsPbI_3_ volume and its initial PL wavelength maximum. Lower concentrations of smaller CsPbI_3_ are predicted to have the most accurate and sensitive assay response. From an end-user perspective, the size and concentration of CsPbI_3_ are required, and combined with the reaction conditions (*e.g.*, concentrations), the appropriate amount of CsPbI_3_ required for optimum response can be determined. Finally, it is also worth considering CsPbI_3_ with more nanoplatelet-like morphologies^50,51^ or nanoparticles that start from mixed halide concentrations that may provide more stability and shelf life.^31^ Ultimately, this new type of colorimetric assay for organic reactions could find utility at the benchtop, as a quantitative PL-based analogue to thin-layer chromatography (TLC) or, at industrial scales, as an in-line detector for flow chemistry.

## Conclusions

In this study, well-characterized and quantified CsPbI_3_ nanoparticles were halide exchanged with Br^–^ ions forming CsPbBr_*x*_I_3–*x*_, and the resulting optical blueshifts were systematically quantified. The determination of the sensitivity factor (*C*) relates wavelength shift per micromolar quantities of Br^–^ ions in solution, as a function of the starting CsPbI_3_ concentration. This then allowed for the colorimetric monitoring of an organohalide reaction where the Br^–^ byproducts were detected in solution. The utility of this assay was then shown by the ease of which a solvolysis reaction kinetics could be monitored by the PL wavelength change. Rate constants, equilibrium, and activation energies of the external reaction were determined, resulting in values close to those reported in the synthetic literature, and determined by techniques like NMR. Future assays will explore how to incorporate PL intensity data into the assay, as well as monitor other halides and organohalide reactions using different CsPbX_3_ compositions and morphologies.

## Supporting Information Available

Tables S1 and S2; additional Figures S1–S5 in the form of TEM micrographs, PL response raw data, and CsPbI_3_ volume calculations (PDF)
