# Supplementary material for: Using the Photoluminescence Color Change in Cesium Lead Iodide Nanoparticles to Monitor the Kinetics of an External Organohalide Chemical Reaction by Halide Exchange
Source: ACS Nanosci Au. 2023 Sep 7;3(5):418–23. doi: 10.1021/acsnanoscienceau.3c00026 (PMC10588436; doi:10.1021/acsnanoscienceau.3c00026)
Supplement: Supplementary file 1 — ng3c00026_si_001.pdf [file ng3c00026_si_001.pdf]

Supporting Information

Using Photoluminescence Color Change in Cesium Lead Iodide Nanoparticles to Monitor the Kinetics of an External Organohalide Chemical Reaction by Halide Exchange

Tennyson L. Doane[†], Kevin J. Cruz, Tsung-Hsing Chiang, Mathew M. Maye*

Department of Chemistry, Syracuse University, Syracuse New York 13244 U.S.A.
[†]Present Address: Department of Chemistry, LeMoyne College,
Syracuse, NY, 13214, U.S.A.*mmmaye@syr.edu*

Supporting Tables:

Table S1: Exponential Fits for the Solvolysis Data for t = 0-150 minutes

T °C		A mM	B mM	C 1×10^{-6} s^{-1}	D mM	E 1×10^{-6} s^{-1}	Correlation
50	$y = A + Be^{(-Ct)}$	1.17	8.81	82.53	-	-	0.998
55	$y = A + Be^{(-Ct)}$	-0.28	10.28	106.68	-	-	0.999
60	$y = A + Be^{(-Ct)}$	-0.18	10.61	252.12	-	-	0.985
65	$y = A + Be^{(-Ct)}$	1.65	8.63	1059.30	-	-	0.974
70	$y = A + Be^{(-Ct)}$	1.46	8.82	780.5	-	-	0.972
70	$y = A + Be^{(-Ct)} + De^{(-Et)}$	0.00	7.88	923.1	2.46	77.9	0.975
70*	$y = A + Be^{(-Ct)}$	1.83	8.55	887.62	-	-	

* 0-3600s

Table S2: Calculated rates for sovolysis (*k1*) and acid catalyzed ether cleavage (*k2*)

T °C	*k1* 1×10^{-6} s^{-1}	k2 1×10^{-6} s^{-1}
50	82.53	-
55	106.68	-
60	252.12	-
65	884.70	174.60
70	725.40	162.22

Supporting Figures:

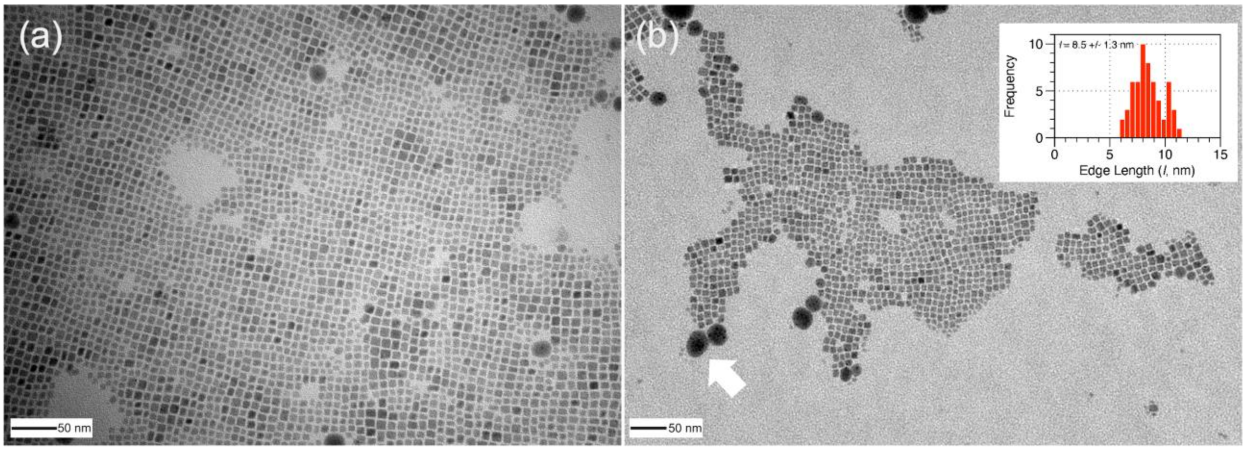

Figure S1: Representative TEM micrographs of OAm- and OAc-caped $CsPbI_3$ directly after synthesis (a), and after aging (b) with larger platelets and spherical $CsPb_2I_5$ phases visible (arrow) (b). Insert: Representative histogram of $CsPbI_3$ edge lengths (*l*) from (a), resulting in *l* = 8.5 +/- 1.3 nm.

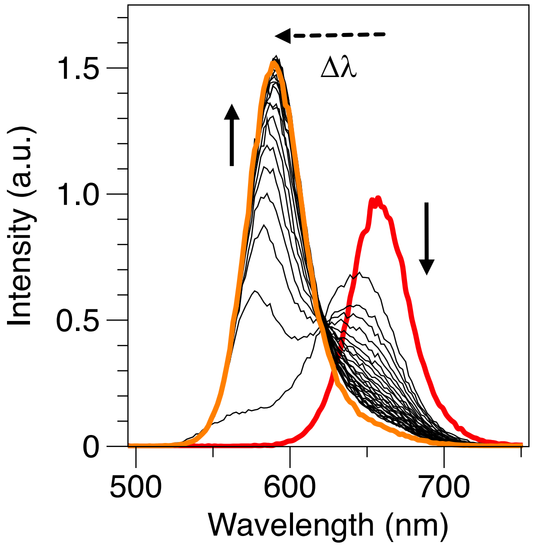

Figure S2: A representative data set monitoring HE at 15s intervals over 200s total ([$CsPbI_3$] = 44 nM, [HBr] = 92 μM).

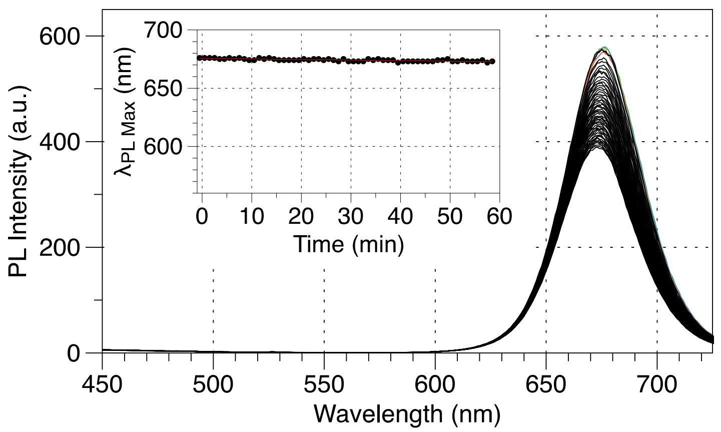

Figure S3: Control experiment following PL wavelength of $CsPbI_3$ in the presence of 79.4 µM 2-bromo-2-methylbutane (S) in hexanes over the course of an hour at 25°C.

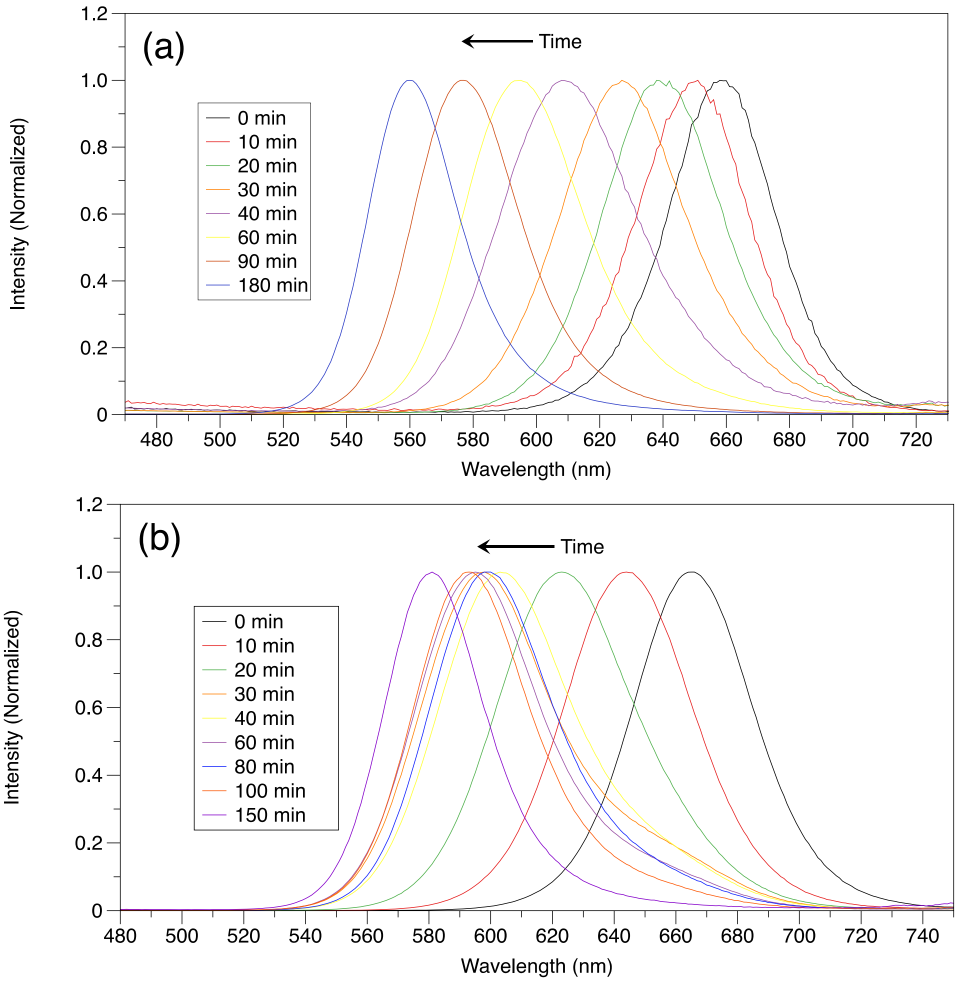

Figure S4: Representative PL spectra for the $CsPbI_3$ assay of the reaction at 60 (a) and 70 °C (b).

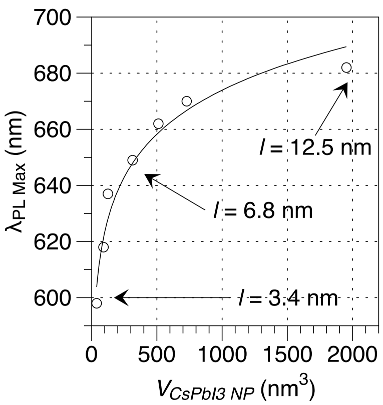

Figure S5: (a) Calculated relationship between PL maximum (λ_{PLMax}) and $CsPbI_3$ volume for cube $CsPbI_3$ morphologies, based on data reported in reference[S1].

Supporting References

(S1) Swarnkar, A.; Marshall, A. R.; Sanehira, E. M.; Chernomordik, B. D.; Moore, D. T.; Christians, J. A.; Chakrabarti, T.; Luther, J. M. Quantum Dot-Induced Phase Stabilization of -CsPbI3 Perovskite for High-Efficiency Photovoltaics. *Science* **2016**, *354* (6308), 92–95. https://doi.org/10.1126/science.aag2700.
